# Rhabdomyolysis in a Young Patient due to Hypothyroidism without Any Precipitating Factor

**DOI:** 10.1155/2019/4210431

**Published:** 2019-12-03

**Authors:** Dhineshreddy Gurala, Kartikeya Rajdev, Roshan Acharya, Pretty Sara Idiculla, Saad Habib, Michael Krzyzak

**Affiliations:** ^1^Resident, Internal Medicine, Staten Island University Hospital, Northwell Health, Staten Island, NY, USA; ^2^Foreign Medical Graduate, Sree Gokulam Medical College and Research Foundation, Alamthara, India; ^3^Hospitalist, Internal Medicine, Staten Island University Hospital, Northwell Health, Staten Island, NY, USA

## Abstract

Hypothyroidism is characterized by decreased hormone production, which results in various clinical manifestations in different organ systems. Muscular symptoms are common in patents with clinical hypothyroidism which includes muscle cramps, myalgia, and mild to moderate elevation of creatinine kinase less than five times the upper limit of normal. However, rhabdomyolysis due to hypothyroidism is rare and in most of the reported cases a precipitating factor has been found. We report a unique case of a 35-year-old male with no past medical history who presented with rhabdomyolysis due to newly diagnosed hypothyroidism without any precipitating factors and was treated successfully with intravenous fluids and levothyroxine.

## 1. Introduction

Hypothyroidism is found in 4.6% of the U.S. population, with 0.3% presenting clinically and 4.3% being subclinical [1]. Muscle involvement in hypothyroidism is common, involving 79% of patients [2]. Muscular manifestations range from muscle weakness, myalgia, muscle cramps with mild to moderate elevation of creatinine kinase to more severe such as Hoffman's syndrome or rhabdomyolysis [3, 4]. Rhabdomyolysis due to skeletal muscle necrosis in hypothyroidism is rare [5, 6] and usually associated with precipitating factor such as statin medication or severe exercise, illicit drugs. However, without any obvious precipitating factors, the incidence of rhabdomyolysis is infrequent [3]. Here in we report a unique case of rhabdomyolysis in young male due to hypothyroidism without any associated precipitating factors.

## 2. Case Presentation

A 35-year-old male with no significant medical history presented to the emergency department with diffuse muscle pain for one week prior to presentation. It initially started in his neck which progressed to his shoulder, trunk, and bilateral lower extremities and was associated with excessive fatigue and recent weight gain of 4.54 kg over two weeks. Review of systems was negative for dry skin, thinning of hair, fever, chills, upper respiratory tract infection, weakness in upper or lower extremities, double vision, changes in memory, or puffy skin. He denied the use of illicit drugs, alcohol abuse, herbal medications, trauma or prolonged immobilization, and did not report any family history of thyroid disease. His vitals were stable with a heart rate of 70 beats per minute, and blood pressure was 130/80 mm Hg. Physical examination was positive for diffuse mild muscle tenderness with normal 5/5 motor strength in upper and lower extremities. Electrocardiography showed normal sinus rhythm. Laboratory examination revealed normal hemogram and serum electrolytes including phosphate and calcium levels as shown in the Table 1. His serum creatinine was 1.2 mg/dl (baseline creatinine is 1.0−1.2). He had elevated serum creatinine kinase (CK) of 11760 U/L and elevated lactate dehydrogenase (LDH) of 544 U/L. His lipid profile was deranged with total cholesterol of 240 mg/dl, triglyceride of 284 mg/dl, and LDL-cholesterol of 138 mg/dl. His urine was bloody in appearance, and urine analysis was negative for erythrocytes but was positive for urine myoglobin.

The patient was admitted to the hospital for rhabdomyolysis and was started on intravenous fluids. He was found to have an TSH (Thyroid Stimulating Hormone) of 100 micro IU/ml with a low free T4 level of 0.5 micro g/dl (Table 1). He was diagnosed with hypothyroidism and treatment with levothyroxine was initiated. Further work-up including Rheumatoid Factor (RF), Cyclic Citrullinated Peptide (CCP) Antibody, Antinuclear Antibody (ANA), Erythrocyte Sedimentation Rate (ESR), C-Reactive Protein (CRP), ds DNA, Coombs test, HIV, and urine drug screen was normal. Our patient showed significant improvement in his symptoms and the CK levels went down to 7600 *µ*/l with hydration by the third day of hospitalization. He was eventually discharged home on the fourth day of hospitalization and followed up as an out-patient.

## 3. Discussion

Hypothyroidism causes a broad spectrum of clinical manifestations such as generalized fatigue, dry skin, constipation, bradycardia, hypothermia, diastolic hypertension, and pericardial effusion. Muscular symptoms range from muscle weakness, stiffness, myalgia, muscle cramps to severe rhabdomyolysis, and pseudohypertrophy of the muscles (Hoffman's syndrome) [3, 4].

Rhabdomyolysis is defined by skeletal muscle necrosis and release of intracellular muscle contents into the circulation. Common causes of rhabdomyolysis include seizure, trauma, medications such as a statin, illicit drugs (methadone), alcohol, strenuous exercise, inflammatory myopathies (polymyositis), electrolyte abnormalities (hyponatremia, hypokalemia, hypocalcemia), and some of the glycogen storage disorders [7]. Rarely, it may also develop in patients with hypothyroidism [8, 9]. The pathophysiology of rhabdomyolysis in hypothyroidism is still unclear. Myolysis is attributable to changes in muscle fibers from fast twitching to slow twitching muscle fibers, deposition of glycosaminoglycans, poor contractility of actin-myosin units, low myosin ATPase activity, or low ATP turn over in the skeletal muscles [10]. Some of the possible explanations for these are inhibition of mitochondrial oxidative phosphorylation results in reduced ATP production and dysregulation of metabolic pathways such as the Kreb's cycle, fatty acid catabolism, and glycolytic energy production [11].

Diagnosis of rhabdomyolysis is based on the elevation of creatinine kinase up to five times the standard limit. Our patient had 50 times the usual limit of CK, had clinical symptoms of hypothyroidism such as weight gain, extreme fatigue, and laboratory examination showed high TSH, low T4 and dyslipidemia. Our patient did not have any other risk factors for the development of rhabdomyolysis and his work-up was negative for infectious, inflammatory, or autoimmune etiology. These findings led to the diagnosis of hypothyroidism induced rhabdomyolysis.

Initial management of rhabdomyolysis consists of intravenous fluids administration and to correct electrolyte abnormalities such as hyperkalemia, hyperphosphatemia, hypocalcemia, hyperuricemia, and metabolic acidosis. Acute kidney injury (AKI) is one of the most common complications with the reported frequency of AKI ranges from 15 to over 50% [12, 13]. Early and aggressive fluid resuscitation is necessary to prevent acute kidney injury [12]. The type of fluid and rate of repletion are unclear, however, initial fluid resuscitation with isotonic saline at the rate of 100 to 200 ml/hour is suggested to maintain or enhance renal perfusion, thereby minimizing ischemic injury. Sequential monitoring of CK levels, volume status of the patient, and urine output are needed to monitor response to the treatment and to adjust the rate of intravenous fluids. Fluid repletion should be continued until CK levels decreases to <5000 U/l and continue to fall. Measurement of TSH and free T4 every 4−6 weeks is necessary to assess the response. If TSH remains above the reference range, the dose can be increased by 25 to 50 *µ*g/day until therapeutic goal has been achieved.

We suggest to keep hypothyroidism in one of the differential diagnosis in patients with rhabdomyolysis. According to the American Thyroid Association clinical guidelines, an increase in serum concentration of either CK or LDH for at least two weeks is enough to screen patients for hypothyroidism [13]. Our case illustrates the importance of screening for hypothyroidism in patients with rhabdomyolysis, and symptoms of hypothyroidism in the absence of other etiologies [14, 15].

## 4. Conclusion

Rhabdomyolysis is a rare but potentially severe complication of hypothyroidism. Clinicians should be aware of this unique possibility and be cautious in patients presenting with symptoms of hypothyroidism and muscular manifestations. It is necessary to screen patients with elevated muscle enzymes for hypothyroidism, as recommended by the American Thyroid Association. Early diagnosis and prompt treatment of hypothyroidism induced rhabdomyolysis are essential in preventing grave consequences.

## Figures and Tables

**Table 1:** Laboratory results.

Variable	Reference range	Day 1	Day 2	Day 3
Hemoglobin (mg/dl)	14–18	15.6		
WBC count (K/*µ*l)	4.8–10.8	7.84		
Platelets (K/*µ*l)	130–400	206		
Glucose random (mg/dl)	70–99	94		
Serum sodium (mmol/litre)	135–146	137	138	141
Serum potassium (mmol/litre)	3.5–5.0	4.3	3.8	3.8
Serum chloride (mmol/litre)	98–110	96	97	101
Anion gap	7–14	15	13	14
BUN (mg/dl)	10–20	14	11	9
Serum creatinine (mg/dl)	0.7–1.5	1.2	1.3	1.2
Calcium (mg/dl)	8.5–10.1	9.3	8.7	8.6
Magnesium (mg/dl)	1.8–2.4			1.9
TSH (*µ*IU/ml)	0.27–4.2	111	100	
T4 (*µ*g/dl)	4.6–12		0.5	
LDH (U/l)	50–242	544		
CK (U/l)	0–225	9553	11760	7600
CRP (mg/dl)	0–0.4		0.27	
RF (IU/ml)	0–13		<10	
CCP (units)	0–19		<8	
Ds DNA (IU/ml)	≤29		<12	
ESR (mm/hr)	0–10		3	
TG (mg/dl)	40–150	284		
HDL-cholesterol (mg/dl)	40–60	29		
LDL-cholesterol (mg/dl)	50–100	138		
Total-cholesterol (mg/dl)	100–200	240		
